# Knowledge, Attitudes, and Behaviors Towards Cyberchondria: A Cross-Sectional Study Among Italian Adolescents

**DOI:** 10.3390/children13060736

**Published:** 2026-05-26

**Authors:** Maria Catone, Vincenza Sansone, Giorgia Della Polla

**Affiliations:** Department of Experimental Medicine, University of Campania “Luigi Vanvitelli”, Via Luciano Armanni 5, 80138 Naples, Italy; maria.catone@unicampania.it (M.C.); vincenza.sansone@unicampania.it (V.S.)

**Keywords:** cyberchondria, adolescents, internet, survey, Italy

## Abstract

**Highlights:**

**What are the main findings?**

- Beyond demographic and health-related factors, attitudinal variables independently predicted both online health information-seeking and cyberchondria severity.
- Family-related factors, including lower paternal educational level and absence of parental chronic conditions, were also associated with higher cyberchondria, suggesting a role for parental mediation in adolescents’ digital health behaviors.

**What are the implications of the main findings?**

- Prevention strategies should move beyond knowledge-based approaches and also target beliefs that may drive and reinforce excessive online searching.
- Healthcare professionals should be trained to identify cyberchondria in adolescents and guide them towards reliable digital resources.

**Abstract:**

**Background/Objectives**: Cyberchondria is the compulsive and repetitive search for health-related information online. Adolescents may be vulnerable to cyberchondria due to extensive Internet use and developing health literacy skills; however, evidence from Italy remains limited. The aim of this study is to examine knowledge, attitudes, and behaviors related to cyberchondria among Italian adolescents aged 10–19 years and identify the associated factors. **Methods**: This cross-sectional investigation was conducted from January to March 2025 among adolescents in Naples, southern Italy. Data collection relied on a self-administered structured questionnaire, including the Cyberchondria Severity Scale-12 (CSS-12). Two multivariate logistic regression models identified independent predictors of online health information-seeking and high cyberchondria (CSS-12 ≥ 32 points). **Results**: Among 793 participants (mean age 15.8 ± 2.2 years; 50% female), 59.7% used the Internet to seek health information, and this behavior was independently associated with older age, female gender, lower self-perceived health, chronic conditions, and increasing CSS-12 values. The mean CSS-12 score reached 31.2 ± 8.3, and 53.4% of participants scored ≥ 32 points. Higher cyberchondria was associated with believing that the Internet is useful for health decision-making, needing more information about cyberchondria, needing more information about a health topic, perceiving the Internet as important for health resources, having a father with a high school diploma or less, and having parents without underlying chronic medical conditions. **Conclusions**: Italian adolescents commonly use the Internet to seek health information, and they present non-negligible cyberchondria levels. This study’s findings emphasize the need for targeted educational interventions promoting safe and critical online health information-seeking behaviors.

## 1. Introduction

Internet use for health-related purposes has emerged as a growing public health issue. Health emergencies such as the COVID-19 pandemic induced individuals to increasingly turn to web-based resources to satisfy health information queries [1]. However, the “infodemic”, the overwhelming proliferation of unreliable information, negatively affected health behaviors and mental health outcomes [2,3]. This highlights the paradoxical nature of Internet use in healthcare, as ease of access to health information is often accompanied by increased risk of exacerbating health-related anxiety [4]. This concept is known as “cyberchondria”, characterized by repetitive and compulsive searching for health information online that, instead of providing reassurance, leads to worsening anxiety and further negative consequences [5]. The UK press created the name in the mid-1990s by combining the terms “cyber” and “hypochondriasis” [5,6]. Then, research by White and Horvitz [7] characterized it as the escalation of medical concerns arising from web-based symptom searches, marking a landmark in this field [8]. Since then, significant scholarly effort has refined and debated its conceptualization [5,8,9]. Among the proposed frameworks, the multidimensional conceptualization is among the most widely adopted in the literature and has been most operationalized in validated measurement tools [10,11,12]. Within this framework, cyberchondria encompasses four core dimensions: excessiveness, characterized by repetitive and escalating online health searches; compulsion, referring to the degree to which such searches interfere with daily offline and online functioning; distress, involving the emotional discomfort triggered by health-related information encountered online; and reassurance, namely the tendency to seek professional medical advice as a result of findings discovered through online searches [5,10]. Cyberchondria is consistently associated with heightened anxiety, depression, and reduced quality of life [13,14,15]. At a behavioral level, it may reinforce avoidance of medical care or, conversely, drive unnecessary healthcare-seeking and repeated consultations with healthcare professionals [16]. These patterns hold the potential to place a significant burden on health systems, contributing to inappropriate use of diagnostic resources and reducing the quality of the patient–provider relationship [16,17].

Against this background, research on cyberchondria among adolescents has gained attention, as the implications of online health information-seeking behaviors in this population are increasingly recognized [13,18,19]. Adolescence represents a critical window for intervention, as health behaviors and habits formed during this period tend to consolidate and persist into adulthood [20]. Moreover, the increasing reliance on web-based media as sources of health information has increased adolescents’ exposure to unfiltered and potentially misleading health content, making this age group a priority target for preventive action [21,22]. The ease of access to unfiltered health information may cause those with elevated health anxiety or perceived stress to engage in compulsive searching behaviors, thereby increasing their susceptibility to cyberchondria [23] and leading them to adopt risky behaviors [24,25]. Adolescents represent a particularly susceptible group, given their ongoing developmental maturation, cognitive–emotional immaturity, and high engagement with digital media [20,21]. For example, the study by Akdogan et al. [24] showed that cyberchondria was associated with higher eating disorder risk and diminished body image satisfaction among adolescents, suggesting that this phenomenon may have both psychosocial and clinically relevant consequences in younger populations. Additionally, these dynamics may interfere with the development of healthy help-seeking behaviors, potentially establishing maladaptive patterns [20,23].

Notably, Italian adolescents represent an under-studied population. While research on cyberchondria has expanded internationally, documenting its associations with smartphone addiction, pandemic-related anxiety, and mental health outcomes in adolescents and young adults [13,18,19,26], evidence from the Italian adolescent context remains scarce [14,15].

To address this gap, this cross-sectional study aimed to examine knowledge, attitudes, and behaviors toward online health information-seeking and cyberchondria among a sample of Italian adolescents aged 10–19 years. Specifically, the study aims to (1) examine adolescents’ cyberchondria severity using the CSS-12 and (2) identify the factors independently associated with online health information-seeking behavior and cyberchondria severity among them. The findings from this study can inform evidence-based preventive strategies and public health interventions tailored to the Italian adolescent population and contribute to the growing literature on cyberchondria in youth.

## 2. Materials and Methods

### 2.1. Setting and Survey Population

The present cross-sectional survey was conducted as part of a broader research project aimed at investigating knowledge, attitudes, and behaviors towards eHealth literacy and cyberchondria among adolescents aged 10–19 years [27]. Data were collected between January and March 2025 across 2 middle schools and 5 high schools randomly selected in Naples, southern Italy. The sample was selected using two-stage cluster sampling. Schools were first selected using simple random sampling from an official list of educational institutions in the Campania Region.The classrooms within sampled schools were randomly selected, and adolescents were invited to participate. Students were eligible if they were aged 10–19 years, they were able to complete the questionnaire, and, if minors, they had parental or guardian consent. Sample size calculation was performed assuming a 70% prevalence for the primary outcome “perceiving having sufficient eHealth literacy” [28,29], a 5% margin of error, a 95% confidence level, and a 60% expected response rate, requiring 538 students.

### 2.2. Data Collection

Data collection procedures are presented elsewhere [27]. Participants were informed of the voluntary nature of their participation, data anonymity, confidentiality safeguards, and the scientific purpose of the study. To enhance the response rate, multiple reminders were implemented through direct engagement with teachers. No compensation was provided. Ethical approval for both the research protocol and the survey instrument was granted by the Ethics Committee Campania 2 (protocol number 0032325/i, 18 December 2024).

### 2.3. Survey Instrument

For this survey, an ad hoc questionnaire was developed based on previous research [27]. The self-administered questionnaire was structured in four thematic sections, with an estimated completion time of approximately 30 min. The first section collected sociodemographic and clinical background information, including age, gender, nationality, school grade, cohabitants, chronic medical conditions, and self-perceived health status. Additionally, information regarding adolescents’ parents was collected (educational level, employment, and chronic medical conditions). The second section examined adolescents’ knowledge regarding cyberchondria by asking if they had ever heard about it. Those who answered “yes” were asked to identify the source through which they had encountered it, choosing from a predefined list including personal networks (relatives/friends), digital sources (Internet, social media), healthcare professionals, traditional media (TV, newspapers, radio), institutional bodies (e.g., Ministry of Health), school, or other. The third section assessed respondents’ attitudes and behaviors towards Internet use for health information and their cyberchondria levels through the Cyberchondria Severity Scale-12 (CSS-12) [10]. The CSS-12 is a validated tool designed to assess distress levels and behavioral tendencies linked to compulsive online health information-seeking. This scale includes 12 items categorized into four distinct domains: excessiveness, which is the repetitive or escalating nature of online searches; compulsion, which is associated with the extent to which such behavior interferes with daily functioning both online and offline; distress, being the emotional discomfort triggered by the searches; and reassurance, which is the tendency to seek professional medical advice as a result of information found online [10]. Responses are provided on a 5-point Likert scale (1 = completely disagree; 5 = completely agree), yielding a total score between 12 and 60. The CSS-12 demonstrated good psychometric properties in the original validation study, including good to excellent internal consistency, convergent and discriminant validity, and factorial validity [10]. Italian validation studies confirmed its applicability in the local context [30,31]. In the present sample, the scale showed good internal consistency (Cronbach’s α = 0.82). For this study, participants were categorized into two groups based on the sample’s median CSS-12 score: a high-cyberchondria group (score ≥ 32 points) and a low-cyberchondria group (score < 32 points) [32,33]. Attitudes towards Internet use for health information-seeking were assessed through two items measuring perceived usefulness of the Internet in supporting health-related decisions and perceived importance of being able to access health resources online, both rated on a five-point Likert scale (1 = not at all useful/important; 5 = very useful/very important). Health information-seeking behaviors were explored by asking whether participants used the Internet (yes/no), their average daily online time (less than 1 h/1–2 h/2–3 h/3–4 h/more than 4 h), and whether they had searched for health information online in the preceding three months (yes/no). Depending on their answer, they were prompted to choose from a predefined list of reasons for seeking or not health information online. The fourth part of the questionnaire explored tools and sources used for accessing health information. Respondents were asked to indicate which electronic devices they used for online health searches (e.g., computer, tablet, mobile phone, other, or none), and if they used web-based sources for health information, for example, institutional sources, scientific journals, or others. Finally, they were asked whether they felt the need for additional information on cyberchondria, responding with “yes” or “no”.

### 2.4. Statistical Analysis

All statistical procedures were carried out using STATA 19 [34]. Initially, descriptive statistics including frequency distributions, means, ranges, and standard deviations, were computed to describe the knowledge, attitudes, and behaviors of the sample. Next, univariate analyses were conducted using the chi-square test for categorical variables and Student’s *t*-test for continuous ones, to examine their associations with the outcomes of interest. Lastly, variables reaching a *p*-value ≤ 0.25 at this stage were included in two distinct multivariate logistic regression models to identify those independently linked to the outcomes of interest: having used the Internet in the last three months for health information-seeking (no = 0; yes = 1) (Model 1); CSS-12 score, which was dichotomized into low (32 < points = 0) and high (≥32 points = 1) cyberchondria (Model 2). The independent variables examined in relation to both outcomes included: believing in the internet importance to access health resources online (score <4 = 0; score ≥4 = 1), perceived usefulness of the Internet for health decision-making (score <4 = 0; score ≥4 = 1), and needing additional information on cyberchondria (no = 0; yes = 1). Then, additional independent variables were tested for Model 1; these were: age in years (continuous), gender (male = 0; female = 1), having a parent employed in the health sector (no = 0; yes = 1), presence of a chronic medical condition (no = 0; yes = 1), and CSS-12 score (continuous). For Model 2, independent variables additionally tested included: father’s educational level (high school degree or less = 0; baccalaureate/graduate degree = 1), school grade (middle school = 0; high school = 1), having used the Internet in the last three months for health information-seeking (no = 0; yes = 1), having parents affected by a chronic medical condition (no = 0; yes = 1), and needing more information about a health topic (no = 0; yes = 1). The inclusion and exclusion thresholds for the final multivariate models were set at *p* ≤ 0.20 and *p* > 0.40, respectively. The findings from both logistic regression models were reported as odds ratios (ORs) with associated 95% confidence intervals (CIs). All tests were two-sided, with a significance threshold set at *p* ≤ 0.05. To handle missing data, listwise deletion was applied, and no imputation method was adopted.

## 3. Results

Out of 1157 students approached, 793 completed the questionnaire, yielding a response rate of 68.5%. Sociodemographic and clinical characteristics of the sample are summarized in Table 1.

### 3.1. Sociodemographic and Anamnestic Characteristics

The sample showed balanced gender distribution with a mean age of 15.8 years (±2.2). Only 1.8% were non-Italian. A minority attended middle school (14.9%) and lived with at least three family members (3.3 ± 1). Regarding parental education, 25.8% of mothers and 17.3% of fathers held baccalaureate or university degrees. Most parents were employed, with fathers having higher employment rates (97.2%) than mothers (53.2%). A small percentage of parents worked in the health sector (8.5%). Participants’ mean self-perceived health score was 8.5 ± 1.3 (scale 1–10). Only 9.7% had chronic conditions, most commonly respiratory/allergic (29.3%) and endocrine disorders (16%), followed by sensory organ (9.3%), cardiac and hematological (9.3%), gastrointestinal and urinary (9.3%), and mental health and neurological disorders (8%). Similarly, 11.8% reported parents with chronic conditions, mainly cardiopulmonary diseases (30.4%), diabetes (23.9%), and hypothyroidism (19.6%), followed by chronic systemic diseases (13%), chronic inflammatory bowel diseases (6.5%), and autoimmune diseases (4.4%).

### 3.2. Adolescents’ Knowledge of Cyberchondria

More than two-thirds (69.8%) of adolescents had no prior awareness of cyberchondria. Among those who knew about it, social media constituted the primary channel of exposure (38.4%), followed by the Internet (35%), and relatives/friends (30.4%). Traditional sources like TV, newspapers, and radio accounted for 15.2%, followed by schools (13.9%) and healthcare professionals (7.2%). Government institutions, including the Ministry of Health, had the least impact (4.2%).

### 3.3. Adolescents’ Online Health Information-Seeking Patterns and Related Attitudes

Adolescents considered the Internet moderately useful for health decision-making (mean 2.7 ± 1.1), while the importance of accessing health resources online scored 2.8 ± 1.2. Only 0.1% reported not using the Internet, 37.8% spent less than three hours online daily, and over half (59.7%) used the Internet for health information-seeking. Moreover, 8.6% struggled to distinguish credible content, 8% found online language difficult, and 4.8% experienced navigation challenges when retrieving health information.

The first multivariate logistic regression model (Model 1, Table 2) identified eight independent predictors of online health information-seeking. Adolescents who were older (OR = 1.27; 95% CI = 1.16–1.39), who were female (OR = 1.87; 95% CI = 1.30–2.70), who had lower self-perceived health status (OR = 0.83; 95% CI = 0.72–0.97), who believed that the Internet is useful for health decision-making (OR = 1.89; 95% CI = 1.09–3.28), who believed the Internet important for accessing health resources (OR = 2.59; 95% CI = 1.62–4.14), who had chronic conditions (OR = 2.07; 95% CI = 1.00–4.27), who had high cyberchondria levels (OR = 1.04; CI = 1.02–1.06), and who needed more cyberchondria information (OR = 1.49; 95% CI = 1.03–2.15) were more likely to seek health information online (Model 1 in Table 2).

### 3.4. Adolescents’ Cyberchondria Levels

The overall mean CSS-12 score was 31.2 ± 8.3 (range 12–55), with 53.4% of the sample scoring ≥ 32 points. The highest mean scores related to the items “I read different web pages about the same perceived condition” (3.4 ± 1.2), “if I notice an unexplained bodily sensation I will search for it on the Internet” (3.3 ± 1.2), and “researching symptoms or perceived medical conditions online leads me to consult with my General Practitioner (GP)” (3.1 ± 1.2) (Table 3). Details of participants’ answers to CSS-12 are described in Table 3.

The second multivariate logistic regression model showed that adolescents who believed that the Internet is useful for health decision-making (OR = 1.95; CI = 1.22–3.11), who needed more information about a health topic (OR = 1.79; CI = 1.15–2.80), who had a father with a high school diploma or below (OR = 0.57; CI = 0.37–0.88), who needed more information about cyberchondria (OR = 1.46; CI = 1.05–2.02), who believed that the Internet is important for accessing health resources (OR = 1.53; CI = 1.04–2.27), and who had parents without any chronic medical conditions (OR = 0.59; CI = 0.36–0.96) were more likely to have a CSS-12 score ≥ 32 points (Model 2 in Table 2).

### 3.5. Adolescents’ Preferred Devices and Sources for Health-Related Online Searches

Only 13% of participants did not rely on electronic devices when seeking health information. Among the remaining majority, smartphones were most used (97%). Computers (14.8%) and tablets (6.1%) were used less frequently. Overall, 91.1% utilized at least one information source. Lastly, nearly half of the sample (45.6%) indicated needing more information about cyberchondria.

## 4. Discussion

This study examined knowledge, attitudes, behaviors, and cyberchondria levels among Italian adolescents between 10 and 19 years of age. To the best of our knowledge, this represents the first investigation of its kind in this population within the Italian context.

Addressing the first aim of the study, our results showed that Italian adolescents present cyberchondria, with demographic, individual, and attitudinal factors predicting both severity and online health information-seeking behavior. The abundance of unverified content, coupled with complex medical terminology, often exceeds their health literacy, creating barriers to understanding [22,35]. Despite these challenges, over half of our sample sought health information online in the preceding three months, and the CSS-12 score results are comparable with recent research conducted on similar populations [26].

Regarding the second aim, the models revealed several variables significantly associated with the outcomes of interest. With respect to online health information-seeking behavior, older adolescents showed a greater likelihood of online health information-seeking, reflecting increasing health awareness and autonomy during this developmental stage [22]. Adolescents become increasingly interested in understanding their bodies, often turning to accessible online resources for privacy and independence [22,36], and generally demonstrate greater Internet proficiency than their younger peers [22]. This developmental trend intersects with observed female predominance in online health-seeking, consistent with prior research [22]. Gender differences may reflect broader sociocultural influences encouraging females toward proactive health-conscious approaches [37]. Participants with lower self-perceived health status were more likely to seek online health information, possibly to understand symptoms, explore solutions, or obtain reassurance, one of the core elements of cyberchondria [10]. Problematic online searching is exacerbated by search algorithms [7], alarming diagnoses, escalating concerns, and ultimately creating self-reinforcing compulsive patterns that are difficult to interrupt [7,38]. Increasing cyberchondria levels predicted adolescents’ online health-seeking behavior, reflecting the self-perpetuating cycle typical of cyberchondria, where searches provide temporary reassurance but increase anxiety and trigger further searching. This feedback loop undermines well-being [16,18], as ambiguous or conflicting information heightens uncertainty and leads to additional searches [39]. Additionally, having chronic conditions doubled the likelihood of using the Internet for health information-seeking, aligning with the existing literature [40], likely because adolescents with chronic illnesses experience ongoing informational needs about treatment, prognosis, and self-management [41,42]. Previous research has shown that attitudes have a relevant impact on Internet use for health information purposes too [42,43]. Attitudinal predictors, such as believing the Internet is important for accessing health resources and believing it is useful for health decisions, were significantly associated with online health-seeking behavior. These findings suggest adolescents with positive Internet attitudes are more inclined to use it for health purposes. Attitudes tangibly influence behavior, directly shaping health-related choices [44]. Therefore, promoting critical evaluation skills may foster constructive attitudes and effective, safe online health information-searching behaviors.

On the other hand, cyberchondria severity was predicted by several variables. Our findings show that adolescents with fathers who have a lower educational background tended to have higher CSS-12 scores. This finding is consistent with a recent study, where adolescents with illiterate fathers were more likely to have higher cyberchondria [26]. Adolescents whose parents have higher educational attainment may benefit from greater parental guidance in critically evaluating online health information, thereby reducing the risk of compulsive and anxiety-driven health searches. Evidence suggests that more educated parents tend to engage in reflective conversations about online content, facilitating a more engaged and constructive oversight of adolescents’ interactions with digital health information [45]. By contrast, parents with lower educational attainment may perceive a generational divide in which younger generations are regarded as more technologically proficient, potentially leaving adolescents without adequate critical guidance when navigating online health resources [27,45]. Additionally, having parents without chronic conditions was associated with higher cyberchondria. One possible reason is that adolescents from families without significant health issues may lack direct healthcare experience and realistic health perspectives, making them more susceptible. In contrast, those with chronically ill parents may have developed appropriate healthcare-seeking behaviors. A similar result appeared in a previous study where sufficient eHealth literacy was associated with not having parents with chronic conditions [27]. However, due to limited comparable data, direct comparisons remain challenging. The significant association between cyberchondria and the belief that the Internet is useful for health decision-making, as well as the perceived importance of accessing online health resources, suggests that positive attitudes toward online health information-seeking may function as behavioral amplifiers. Notably, these attitudinal variables independently predicted both the likelihood of engaging in online health information-seeking and higher cyberchondria severity, suggesting a dual role: they may initiate the behavior and sustain its escalation toward problematic levels. In line with the metacognitive framework proposed by Fergus and Spada [46,47], such beliefs may operate similarly to positive metacognitive beliefs, possibly working as implicit convictions that engaging in online health searches is a necessary, protective, and adaptive strategy. Rather than reducing health-related distress, these beliefs may sustain and reinforce information-seeking behavior, ultimately contributing to higher cyberchondria severity. Moreover, adolescents who needed more information about a health topic were more likely to present CSS-12 scores ≥ 32 points, suggesting that unmet informational needs may represent a trigger in the development of cyberchondria: when individuals perceive gaps in their knowledge, they are more prone to turning to the Internet for health-related searches, which may escalate toward more severe concerns and increased anxiety [7]. Such patterns are consistent with models of reassurance-seeking, where attempts to reduce uncertainty paradoxically maintain and amplify distress [48]. Finally, the association with the need for more information about cyberchondria suggests that adolescents experiencing these symptoms recognize their problematic patterns but lack sufficient knowledge about the condition itself, indicating an opportunity for targeted educational interventions. Specifically, eHealth literacy may mitigate this process, as individuals with stronger abilities in retrieving, assessing, and managing online health information may carry a lower risk of maladaptive reassurance-seeking and cyberchondria [49].

The results of this study yield meaningful insights with practical repercussions for clinical settings and health promotion initiatives. Educational interventions should focus on eHealth literacy training emphasizing critical evaluation of web-based health information, recognition of cyberchondria symptoms and potential consequences, and adequate online health information-seeking strategies. Moreover, when considering attitudes, the findings of this study emphasize the importance of going beyond informational programs and developing actions also focused on the attitudinal layer that may sustain excessive online health information-seeking to prevent cyberchondria among adolescents. Given identified family-related predictors, interventions should consider family-based approaches, helping parents understand their role in educating adolescents about Internet use. Healthcare providers need training to recognize and address cyberchondria symptoms among adolescents, guiding them toward reliable online resources.

When interpreting the present findings, certain limitations must be considered. The use of a cross-sectional approach inherently limits causal attribution. The sampling frame, confined to the urban area of Naples in southern Italy, may not adequately capture the heterogeneity of the broader Italian adolescent population, nor allow comparisons with international samples. Additionally, data derived from self-report instruments carry well-known limitations, including susceptibility to recall bias and the tendency to provide socially acceptable responses, particularly in the case of problematic behaviors. Furthermore, cyberchondria may be shaped by broader social and cultural factors, including socioeconomic conditions, educational systems, social support structures, and cultural attitudes toward health and technology, that have not been assessed in the present study.

On the other hand, this study has interesting strengths. It is among the first to explore cyberchondria in Italian adolescents, addressing a knowledge gap in this context. The relatively large sample and acceptable response rate increase confidence in the findings. The use of the CSS-12 ensured reliable assessment of cyberchondria, and the multivariate approach allowed us to identify independent predictors beyond simple associations.

Future research should employ longitudinal designs to clarify how cyberchondria emerges and evolves during adolescence. Intervention studies examining educational programs are needed to determine evidence-based strategies for raising awareness and preventing cyberchondria among youth. Moreover, qualitative research exploring adolescents’ experiences with online health information-seeking and their perceptions of cyberchondria symptoms could provide deeper insights into the mechanisms underlying the associations observed in quantitative studies. Beyond individual-level factors, future studies should examine how socioeconomic conditions, educational systems, and social support structures may impact the expression and severity of cyberchondria across different cultural contexts, extending research to populations that remain underrepresented in the current literature.

## 5. Conclusions

This study provides evidence on cyberchondria and online health information-seeking behaviors among Italian adolescents. The findings show that adolescents commonly use the Internet for health information-seeking and have non-negligible levels of cyberchondria. Identification of individual, familial, and attitudinal determinants underscores the multifaceted nature of these phenomena and emphasizes the urgent need for comprehensive educational interventions, including from family and schools. Fostering adolescents’ competence in discerning and effectively engaging with digital health information represents a crucial public health priority for maximizing digital health resource benefits while mitigating associated risks, including cyberchondria and possible exposure to misinformation.

## Figures and Tables

**Table 1:** General sample characteristics.

**Adolescents’ Characteristics**	**N**	**Mean (±SD)**	**%**
*Age, in years (10–19)*		15.8 (±2.2)	
*Sex* ^#^			
Male	393		50
Female	393		50
*Italian nationality*			
No	14		1.8
Yes	779		98.2
*School grade*			
Middle school	118		14.9
High school	675		85.1
*Having cohabitants* ^#^		3.3 (±1.0)	
*Chronic medical condition* ^#^			
No	700		90.3
Yes	75		9.7
*Reported conditions* ^#^*			
Respiratory and allergic	22		29.3
Endocrine disorders	12		16
Sensory organ	7		9.3
Cardiac and hematological	7		9.3
Gastrointestinal and urinary	7		9.3
Mental health and neurological	6		8
*Self-perceived health status*		8.5 (±1.3)	
**Parents’ Characteristics**	**N**	**Mean (±SD)**	**%**
*Mother with baccalaureate/graduate degree* ^#^			
No	577		74.2
Yes	201		25.8
*Father with baccalaureate/graduate degree* ^#^			
No	627		82.7
Yes	131		17.3
*Mother being employed* ^#^			
No	341		46.8
Yes	388		53.2
*Father being employed* ^#^			
No	21		2.8
Yes	718		97.2
*Having a parent employed in the health sector*			
No	642		91.5
Yes	60		8.5
*Parent with underlying chronic medical condition* ^#^			
No	685		88.2
Yes	92		11.8
*Reported conditions* ^#^*			
Cardiopulmonary diseases	28		30.4
Diabetes	22		23.9
Hypothyroidism	18		19.6
Chronic systemic diseases	12		13
Chronic inflammatory bowel diseases	6		6.5
Autoimmune diseases	4		4.4

^#^ The totals for each item may not correspond exactly to the overall study population due to missing data; * participants were allowed to select more than one option.

**Table 2:** Results of the multivariate logistic regression analysis showing the predictors of the outcomes of interest.

Variable	OR	SE	95% CI	*p*
Model 1. Use of the Internet for health information-seeking in the preceding three months Log likelihood = −353.81, χ^2^ = 130.32 (9 df), *p* < 0.0001				
Older age	1.27	0.06	1.16–1.39	<0.001
Believing that the Internet is important to access health resources	2.59	0.62	1.62–4.14	<0.001
Female	1.87	0.35	1.30–2.70	0.001
Having high cyberchondria levels	1.04	0.01	1.02–1.06	0.001
Having a low self-perceived health status	0.83	0.06	0.71–0.97	0.020
Believing that the Internet is useful for health decision-making	1.89	0.53	1.09–3.28	0.023
Needing more information about cyberchondria	1.49	0.28	1.03–2.15	0.034
Having a chronic medical condition	2.07	0.76	1.00–4.27	0.049
Having parents employed in the health sector	0.66	0.21	0.35–1.25	0.202
Model 2. Having CSS-12 score ≥ 32 points Log likelihood = −424.76, χ^2^ = 65.17 (7 df), *p* < 0.0001				
Believing that the Internet is useful for health decision-making	1.95	0.47	1.22–3.11	0.005
Needing more information about a health topic	1.79	0.41	1.15–2.80	0.011
Having a father with a high school diploma or below	0.57	0.13	0.37–0.88	0.012
Needing more information about cyberchondria	1.46	0.24	1.05–2.02	0.023
Believing that the Internet is important to access health resources	1.53	0.31	1.04–2.27	0.032
Having parents without a chronic medical condition	0.59	0.15	0.36–0.96	0.033
Having used the Internet for health information-seeking in the last three months	1.45	0.28	0.99–2.10	0.051

**Table 3:** Participants’ CSS-12 mean scores.

Domain	Item	Mean Score ± SD
Excessiveness	1. If I notice an unexplained bodily sensation, I will search for it on the Internet	3.3 ± 1.2
Compulsion	2. Researching symptoms or perceived medical conditions online distracts me from reading news/sports/entertainment articles online	2.3 ± 1.2
Excessiveness	3. I read different web pages about the same perceived condition	3.4 ± 1.2
Distress	4. I start to panic when I read online that a symptom I have is found in a rare/serious condition	2.9 ± 1.4
Reassurance	5. Researching symptoms or perceived medical conditions online leads me to consult with my GP	3.1 ± 1.2
Excessiveness	6. I enter the same symptoms into a web search on more than one occasion	2.8 ± 1.2
Compulsion	7. Researching symptoms or perceived medical conditions online interrupts my work (e.g., writing emails, working on word documents or spreadsheets)	2.1 ± 1.1
Distress	8. I think I am fine until I read about a serious condition online	2.2 ± 1.2
Distress	9. I feel more anxious or distressed after researching symptoms or perceived medical conditions online	2.8 ± 1.3
Compulsion	10. Researching symptoms or perceived medical conditions online interrupts my offline social activities (e.g., reduces time spent with friends/family)	1.9 ± 1.0
Reassurance	11. I suggest to my GP/medical professional that I may need a diagnostic procedure that I read about online (e.g., a biopsy/a specific blood test)	2.3 ± 1.2
Reassurance	12. Researching symptoms or perceived medical conditions online leads me to consult with other medical specialists	2.4 ± 1.2

## Data Availability

The anonymous data presented in this study are available on request from the corresponding author. Access to these data is restricted to safeguard the privacy and confidentiality of the study participants, in accordance with the informed consent terms.

## Abbreviations

The following abbreviations are used in this manuscript:

CI	Confidence Interval
CSS-12	Cyberchondria Severity Scale-12
COVID-19	Coronavirus Disease 2019
df	Degrees of Freedom
GP	General Practitioner
OR	Odds Ratio
SD	Standard Deviation
